# Proteomic Analysis of Saliva Identifies Potential Biomarkers for Orthodontic Tooth Movement

**DOI:** 10.1100/2012/647240

**Published:** 2012-07-31

**Authors:** Mohd Faiz Ellias, Shahrul Hisham Zainal Ariffin, Saiful Anuar Karsani, Mariati Abdul Rahman, Shahidan Senafi, Rohaya Megat Abdul Wahab

**Affiliations:** ^1^School of Bioscience and Biotechnology, Faculty of Science and Technology, Universiti Kebangsaan Malaysia, Selangor, 43600 Bangi, Malaysia; ^2^Institute of Biological Sciences, Faculty of Science, University of Malaya and University of Malaya Centre for Proteomics Research (UMCPR), Universiti Kebangsaan Malaysia, 50603 Kuala Lumpur, Malaysia; ^3^Clinical Oral Biology Department, Faculty of Dentistry, Universiti Kebangsaan Malaysia, 50300 Kuala Lumpur, Malaysia; ^4^Orthodontic Department, Faculty of Dentistry, Universiti Kebangsaan Malaysia, 50300 Kuala Lumpur, Malaysia

## Abstract

Orthodontic treatment has been shown to induce inflammation, followed by bone remodelling in the periodontium. These processes trigger the secretion of various proteins and enzymes into the saliva. This study aims to identify salivary proteins that change in expression during orthodontic tooth movement. These differentially expressed proteins can potentially serve as protein biomarkers for the monitoring of orthodontic treatment and tooth movement. Whole saliva from three healthy female subjects were collected before force application using fixed appliance and at 14 days after 0.014′′ Niti wire was applied. Salivary proteins were resolved using two-dimensional gel electrophoresis (2DE) over a pH range of 3–10, and the resulting proteome profiles were compared. Differentially expressed protein spots were then identified by MALDI-TOF/TOF tandem mass spectrometry. Nine proteins were found to be differentially expressed; however, only eight were identified by MALDI-TOF/TOF. Four of these proteins—Protein S100-A9, immunoglobulin J chain, Ig alpha-1 chain C region, and CRISP-3—have known roles in inflammation and bone resorption.

## 1. Introduction

Orthodontics is an area of dentistry concerning the supervision, guidance and correction of growing and maturing dentofacial structures. Orthodontic treatment is based on the principle that if prolonged pressure is applied to a tooth, it will move as the surrounding bone remodels. This orthodontic tooth movement is characterized by the remodelling of dental and paradental tissues. These tissues include dental pulp, periodontal ligament, alveolar bone, and gingival [1]. Progress of tooth movement can be classified into four stages, that is, activation, resorption, reversal, and restructuring of new bones [2]. An early response to orthodontic force is acute inflammation followed by bone resorption and bone formation. The resorption and formation of bone are due to increments of activities of osteoclast and osteoblast cells [3].

It is always the aim of orthodontists to gain ideal conditions with lower treatment times and fewer appointments. However, when heavy force is applied to hasten tooth movement, the oxygen tension in the periodontium will be compromised due to decreased vascular supply. This will jeopardize the healthy supporting periodontal and alveolar bone structure and may end up with slow progress of the treatment itself. In order to monitor orthodontic tooth movement noninvasively in human beings, changes have been examined in the profile and levels of various enzymes, cytokines, growth factors, and proteoglycans in gingival crevicular fluid (GCF) and saliva. It has been shown that elevated levels of several cytokines, that is, prostaglandin (PG), interleukin (IL)-1*β*, IL-6, epidermal growth factor (EGF) and proteoglycans, in the GCF, are responses to orthodontic force [4–6]. Among components of GCF that change, alkaline phosphatase (ALP), tartrate resistance acid phosphatase (TRAP), lactate dehydrogenase (LDH), and aspartate aminotransferase (AST), have been shown to be potential biomarkers during tooth movement [7–10]. A previous study by Shahrul Hisham et al. [3] showed that ALP, TRAP, and LDH were also present in saliva. This suggested that saliva may also be a source of biomarkers and therefore, a potential diagnostic tool during orthodontic tooth movement. 

The identification of salivary biomarkers and its use as a diagnostic tool has many advantages. It is much easier to collect and sufficient quantities can easily be obtained for analysis. The collection of saliva is also far less invasive compared to other bodily fluids such as GCF, serum and urine. This makes it an attractive option for the study and identification of potential biomarkers. A study looking into the effects of orthodontic treatment on salivary proteins has been performed previously by Zhang et al. using a SELDI-TOF approach [11]. However, this approach looks at relatively low molecular weight proteins and does not determine the identity of these proteins. Only the expression profile is interrogated. Our study aims to identify proteins that change in the saliva during orthodontic tooth movement using a proteomics approach.

## 2. Materials and Methods

### 2.1. Study Population and Sample Collection

This study was carried out with approval from the Research and Ethics committee, Faculty of Dentistry, Universiti Kebangsaan Malaysia, Kuala Lumpur. It is also registered under clinical trial (ISRCTN 47483728), validated by the World Health Organization (WHO) Information sheets were given and informed consent was obtained from all participants.

Saliva samples were obtained from three healthy female subjects (20–25 years old) who were undergoing orthodontic treatment using fixed appliance (0.022′′ × 0.028′′ MBT brackets, ORMCO, USA) at the Orthodontic Specialist Clinic, Universiti Kebangsaan Malaysia, Kuala Lumpur. Unstimulated whole saliva was collected from the subjects at 2.00 pm after 90 minutes of nonoral activities. The subjects were asked to drool out saliva into sterile containers within 10 minutes. Two groups of samples were collected, one taken before application of the 0.014′′ nickel titanium (Niti preformed ORMCO, USA) wire and another sample was taken 14 days after application.

Sampling was performed at day 14 as our previous study showed that TRAP was increased significantly indicating that bone resorption process was active at this time point. Alkaline phosphatase (ALP) and lactate dehydrogenase (LDH) were not significantly different at day 14 suggesting that bone formation is antagonistic to bone resorption and the inflammation process was not active [3]. 

Immediately after collection, a protease inhibitor mix (GE Healthcare Life Science) was added to the saliva. This was followed by centrifugation at 10,000 rpm for 10 min (4°C) to remove insoluble material. Supernatant was then filtered with an acrodisc syringe filter (0.45 *μ*m, Pall Lifescience, USA). Filtered supernatant was then concentrated using Amicon Ultra-4 centrifugal filter units (Millipore, USA) and finally stored at −80°C. Total protein concentration was determined using Bradford assay [12].

### 2.2. First-Dimension Isoelectric Focusing (IEF)

IEF was performed using an EttanIPGphor II isoelectric focusing System (GE Healthcare Bio-Sciences, Uppsala, Sweden). Saliva samples were diluted in buffer containing 7 M urea, 4% (w/v) CHAPS, 2 M Thiourea, 2% (v/v) IPG buffer pH 3–10, 40 mM DTT, and trace amounts of bromophenol blue. Samples were loaded onto rehydrated immobiline DryStrip (isoelectric point [pI] 3–10, 24 cm; GE Healthcare Bio-Sciences, Uppsala, Sweden) performed with EttanIPGphor II Manifold. The proteins were focused by IEF up to 40 kVh. A total of 60 *μ*g protein was loaded to provide the best gel resolution.

### 2.3. Second-Dimension SDS-PAGE

Focused IPG strips were equilibrated for 15** **min in equilibration solution (6** **M urea, 75** **mM Tris-HCl pH 8.8, 29.3% [v/v] glycerol, 2% [w/v] SDS, 0.002% [w/v] bromophenol blue) containing 1% (w/v) DTT for 15 minutes and then alkylated also for 15 minutes in equilibration solution containing 2.5% (w/v) iodoacetamide. Reduced and alkylated IPG strips (containing samples) was applied on top of an SDS-PAGE gel (26** **cm × 20** **cm, 12.5%) and proteins were separated according to their molecular mass using Ettan DALT*six* Large vertical system (GE Healthcare Bio-Sciences, Uppsala, Sweden).

### 2.4. Silver Staining

Following SDS-PAGE, Protein spots were visualized using protocols described in the PlusOne Silver staining kit (GE Healthcare Bio-Sciences, Uppsala, Sweden). The complete protocol was followed for analytical gels. For preparative gels, the protocol was modified so that glutaraldehyde was omitted from the sensitization step and formaldehyde was omitted from the silver reaction step [13]. Silver-stained gels were scanned (UMAX PowerLook 1000 Imaging system) and protein profiles were compared (Image Master Platinum software v 6.0, GE Healthcare Bio-Sciences, Uppsala, Sweden).

### 2.5. Image Acquisition and Analysis

Gel images were analyzed using the Image Master Platinum software v 6.0 (GE Healthcare Bio-Sciences, Uppsala, Sweden). Saliva proteome from the two different groups were compared. A total of nine 2DE gels were run for each group (three gels per subject). This was performed to eliminate experimental and biological variations. To further select against variations between individual samples, the selection criteria for spots were very stringent. Firstly, gels were compared within their respective groups and a representative (virtual) gel was generated. For the generation of the virtual gel, only spots present across all gels within the groups were considered. This was performed to eliminate differences due to interindividual variations. The virtual gels were then compared between the different samples to identify differentially expressed proteins. Statistical analysis was performed by analysis of variance (ANOVA) with significance level set at *P* < 0.05.

### 2.6. Tryptic Digestion

Protein spots were excised and in-gel digested with trypsin (Promega) for mass spectrometric analysis according to published protocols [14–16]. Briefly, excised spots were first destained in destaining solution (15** **mM potassium ferricyanide/50** **mM sodium thiosulphate, 1** **:** **1 [v/v]). The spots were then reduced in a solution containing 10** **mM DTT/100** **mM ammonium bicarbonate for 30** **min at 60°C and alkylated in 55** **mM iodoacetamide/100** **mM ammonium bicarbonate for 20** **min in the dark. The gel pieces were then washed (3 × 20** **min) in 50% acetonitrile/100** **mM ammonium bicarbonate. This was followed by dehydration of the gel pieces with 100% acetonitrile and drying in a vacuum centrifuge (SpeedVac, Thermo Scientific, Savant DNA 120). Subsequently the dried gel pieces were rehydrated with 25 *μ*L of 7** **ng/*μ*L trypsin (Promega trypsin gold) in 50** **mM ammonium bicarbonate buffer and digested at 37°C for 18–20** **h. Tryptic peptides were then extracted using 50% acetonitrile for 15** **min, followed by 100% acetonitrile for 15** **min. The extracted solutions were then pooled into a single tube and dried in a SpeedVac concentrator and solubilized with 10 *μ*L of 10% acetonitrile/40** **mM ammonium bicarbonate.

### 2.7. Mass Spectrometry Identification of Proteins

Protein identification was performed as previously described [17]. Briefly, extracted peptides were first desalted using ZipTip C18 (Millipore, USA) according to protocols described by the manufacturer. The final elution volume following ZipTip cleanup was 1.5 *μ*L. The peptide samples were then mixed (1 : 1) with a matrix consisting of a saturated solution of CHCA (*α*-cyano-4-hydroxycinnamic acid, Sigma) prepared in 50% ACN/0.1% TFA. Aliquots of samples (0.7 *μ*L) were spotted onto stainless-steel sample target plates. Peptide mass spectra were obtained on a MALDI-TOF/TOF mass spectrometer (ABI 4800 plus, Applied Biosystems) in the positive ion reflector mode. For precursor ion selection, all fractions were measured in single MS before MS/MS was performed. For MS/MS spectra, the peaks were calibrated by default. The 20 most abundant precursor ions *per *sample were selected for subsequent fragmentation by high-energy CID. The collision energy was set to 1 keV and air was used as collision gas. The criterion for precursor selection was a minimum S/N of 5. The mass accuracy was within 50 ppm for the mass measurement and within 0.1 Da for CID experiments. The other parameters for searching were of trypsin, 1 missed cleavage, variable modification of carbamidomethyl and oxidation of methionine, peptide charge of 1+, and monoisotopic. For database searches, known contamination peaks such as keratin and autoproteolysis peaks for trypsin were removed before searching. Spectra were processed and analyzed by the Global Protein Server Explorer 3.6 software (Applied Biosystems). This uses an internal MASCOT (Matrix Science, UK) program for matching MS and MS/MS data against database information. The data obtained were screened against human databases downloaded from the Swiss-Prot/TrEMBL homepage (http://web.expasy.org/docs/swiss-prot_guideline.html).

## 3. Results

**Figure 1:**
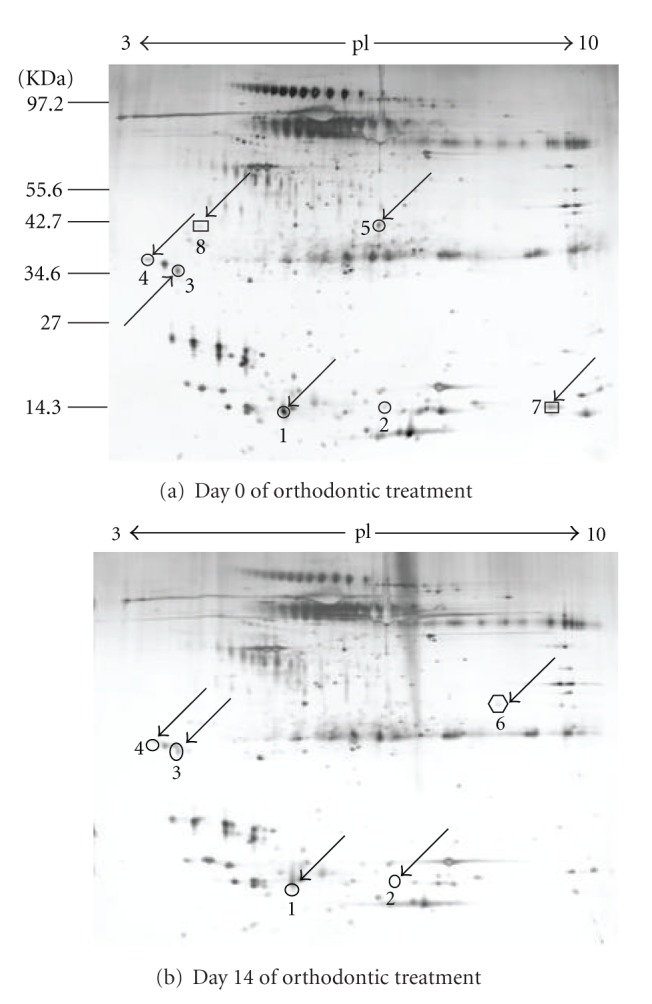
Representative 2DE gels of human saliva proteome. Saliva proteins resolved by 2-DE (pI 3-10, 24 cm). The resulting gels were visualized by silver staining. Arrows indicate protein spots that changed in expression during orthodontic treatment. The numbers correspond to proteins in Table 1.

A sample loading of 60 mg was found to be optimum and resulted in the highest resolution 2DE gels. This resulted in more than 200 individual protein spots being resolved and detected by silver staining. A representative gel is shown in Figure 1.

Following image analysis, nine protein spots appeared to have changed in their expression within the two samples. Eight were successfully identified by MALDI TOF/TOF. The identities of these proteins are shown in Table 1. The protein numbers in Table 1 correspond to the spot numbers in Figure 1. Immunoglobulin J chain isoform (IgJ, two spots identified as IGJ) S100 calcium-binding protein A9 (S100-A9), serum albumin precursor (ALB) and Ig alpha-1 chain C region (IgAC) were down-regulated at day 14 of orthodontic treatment. On the other hand, cysteine-rich secretory protein 3 precursor (CRISP-3) was present only at day 14 while hemoglobin subunit beta (HBB) and 14-3-3 protein *σ* (SFN) were only present at day 0.

## 4. Discussion

Eight proteins were successfully identified using MALDI TOF/TOF. The known functions of these proteins are summarized in Table 2. These proteins have previously been identified in saliva using proteomics approaches [18–20]. However, a change in their expression in relation to orthodontic treatment has never been previously reported. Here we discuss the functions of these proteins and their possible roles in orthodontic tooth movement. 

 Protein S100-A9 (S100-A9) is a calcium-binding protein that functions as a proinflammatory mediator in acute and chronic inflammation. A previous study reported that S100-A9 was involved in osteoblast and chondrocytic maturation, matriceal calcification and was prominent in osteoclasts [21]. The presence of osteoclast indicates an active process of bone resorption. S100-A9 has also been reported to regulate joint inflammation and cartilage destruction during antigen-induced arthritis [22]. This protein is found at high concentration in inflamed tissue [23]. Our study showed an apparent downregulation of S100-A9 protein suggesting that this protein may not be involved during bone resorption in orthodontic tooth movement but is involved in inflammation. This is supported by data from our previous study which showed that at day 14 of treatment, the inflammation process was not active [3]. Thus, downregulation of this protein suggested a suppression of inflammation after 14 days of orthodontic treatment.

Immunoglobulin J chain (IgJ) is a component of IgM or IgA while Ig alpha-1 chain C region (IgAC) is a major immunoglobulin class in body secretions. Both are common components of the immune response in humans. The early phase of orthodontic tooth movement involves acute inflammatory response in the periodontal tissues surrounding the mechanically stressed teeth [24]. Our results showed an apparent downregulation of both IgJ and IgAC after 14 days of orthodontic treatment. Our previous study showed no increase of LDH after 14 days of orthodontic activation [3]. Taken together, this suggested that no further inflammation occurred at this period.

Cysteine-rich secretory protein 3 precursor (CRISP-3) is present in exocrine secretion and in secretory granules of neutrophil. It is believed to play a role in innate immunity [25] and as a potential biological marker for prostate cancer [26]. Our results showed that this protein was present only after 14 days of orthodontic treatment. How it is related to orthodontic tooth movement is unclear.

Serum albumin precursor (ALB) and hemoglobin subunit beta (HBB) are serum proteins. Both proteins have been found to be increased in subjects with periodontal disease [27]. ALB is involved in the regulation of colloidal osmotic pressure of blood and is a major zinc transporter in plasma [28]. HBB is a subunit of hemoglobin which contains four subunits with two alpha and two beta subunits. Each subunit carries an ion-containing molecule (heme) that is involved in oxygen transport from the lung to various peripheral tissues [29]. HBB is usually contained in red blood cells. The function(s) of this protein in saliva is unknown. Our study showed that ALB was present only at day 0 of treatment. Its role(s) in orthodontic tooth movement is also unclear.

An adaptor protein—14-3-3 *σ* also known as epithelial cell marker protein 1 or stratafin (SFN) was found to be present only at day 0. This protein binds to a large number of partners, resulting in the modulation of the activity of the binding partner(s). The loss of protein 14-3-3 *σ* expression has been observed in several types of human cancers, suggesting that it may have a role as a tumour suppressor protein [30]. However, there is no available information on the role of SFN protein in inflammation or bone resorption and formation and the role(s) it may play during orthodontic tooth movement. 

It has to be noted that the number of patients evaluated in our study is relatively small (*n* = 3). Thus, although significant differences were identified, this data should be considered preliminary. Further validation with a much larger sample size will be necessary. It must also be noted that although the expressed proteins could potentially serve as protein biomarkers for the monitoring of orthodontic treatment and tooth movement, the cost of the analyses and the equipment needed may strongly limit the use of proteomics as a diagnostic tool. However, these potential biomarkers can be useful if a different diagnostic technique (such as ELISA) is developed to utilize them. 

## 5. Conclusion

To the best of our knowledge this paper represents a first attempt in using proteomics to identify saliva proteins that change in expression during orthodontic tooth movement. A total of eight proteins were found to have changed in expression. Four of these proteins—Protein S100-A9, immunoglobulin J chain, Ig alpha-1 chain C region, and CRISP-3—have known roles in inflammation and bone resorption. Although these proteins have previously been identified in the saliva, a change in their expression in relation to orthodontic treatment has never been previously reported. Together, these proteins have the potential to be used as potential molecular markers to monitor the progression of orthodontic treatment.

## Figures and Tables

**Table 1:** Summary of saliva proteins with expression change during orthodontic treatment.

Spot no.	Protein name	Accession number (UniProt)	MW (kDa)/pI		Peptides matched/% coverage (Protein score)	Expression change
			Theoretical	Experimental		
1	Protein S100-A9 (S100 calcium-binding protein A9) (Calgranulin-B)	P06702	13.2/5.71	14.5/5.47	4/24% (121)	Downregulated at day 14
2	Serum albumin precursor	P02768	69.3/5.92	14.8/7.00	8/11% (105)	Downregulated at day 14
3	Immunoglobulin J chain	P01591	15.5/4.62	34.0/3.86	10/30% (220)	Downregulated at day 14
4	Immunoglobulin J chain	P01591	15.5/4.62	36.4/3.40	6/27% (198)	Downregulated at day 14
5	Ig alpha-1 chain C region	P01876	37.6/6.08	44.76/6.91	9/20% (260)	Downregulated at day 14
6	Cysteine-rich secretory protein 3 precursor (CRISP-3)	P54108	27.6/8.09	43.25/8.90	2/6% (94)	Present only at day 14
7	Hemoglobin subunit beta (Hemoglobin beta chain) (Beta-globin)	P68871	15.9/6.75	14.83/9.50	6/27% (158)	Present only at day 0
8	14-3-3 protein *σ* (Stratifin) (Epithelial cell marker protein 1)	P31947	27.7/4.68	44.76/4.20	5/22% (147)	Present only at day 0

These are proteins that show statistically significant change (*P* < 0.5) between day 0 and day 14 of orthodontic treatment.

**Table 2:** Summary of identified proteins and their known functions.

Protein	Known function
Protein S100-A9 (S100 calcium-binding protein A9) (Calgranulin-B)	(i) Calcium-binding protein.
	(ii) Promotes phagocyte migration and infiltration of granulocytes at sites of wounding.
	(iii) Plays a role as a proinflammatory mediator in acute and chronic inflammation.

Serum albumin precursor	(i) Good binding capacity for water, Ca^2+^, Na^+^, K^+^, fatty acids, hormones, bilirubin, and drugs.
	(ii) Main function is the regulation of the colloidal osmotic pressure of blood.
	(iii) Major zinc transporter in plasma.

Immunoglobulin J chain	(i) Serves to link two monomer units of either IgM or IgA.
	(ii) Help to bind IgM or IgA to secretory component.

Ig alpha-1 chain C region	(i) Major immunoglobulin class in body secretions.
	(ii) Serve both to defend against local infection and to prevent access of foreign antigens.

Cysteine-rich secretory protein 3 precursor (CRISP-3)	(i) Innate immune response
	(ii) Potential biological marker for prostate cancer

Hemoglobin subunit beta (Hemoglobin beta chain) (Beta-globin)	Involved in oxygen transport from the lung to the various peripheral tissues.

14-3-3 protein *σ* (Stratifin) (Epithelial cell marker protein 1)	(i) Adapter protein.
	(ii) Binds to a large number of partners, generally results in the modulation of the activity of the binding partner.
